# Supplementary figures and images for: Otolith “spawning zones” across multiple Atlantic cod populations: Do they accurately record maturity and spawning?
Source: PLoS One. 2021 Sep 13;16(9):e0257218. doi: 10.1371/journal.pone.0257218 (PMC8437307; doi:10.1371/journal.pone.0257218)

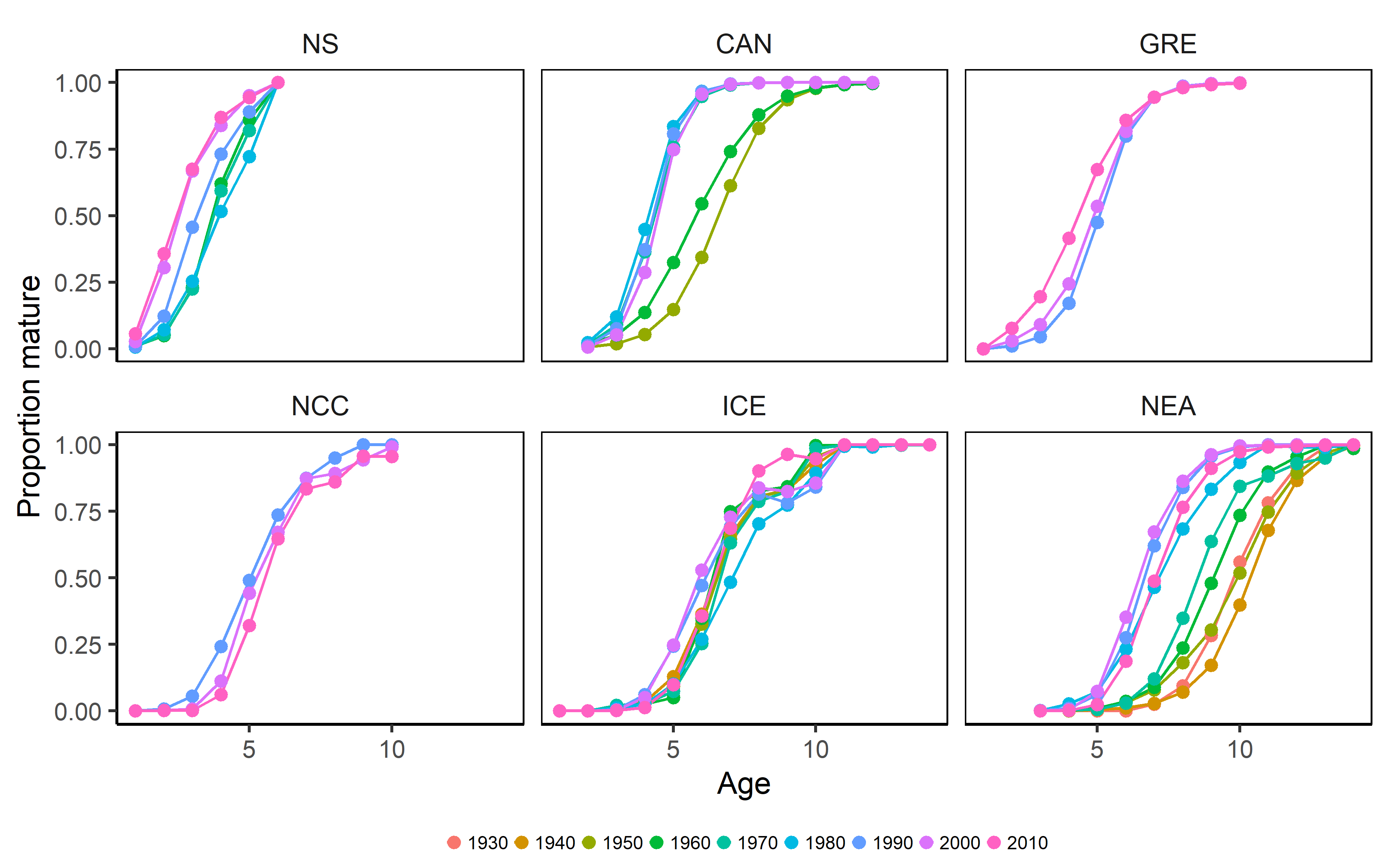

Supplement: S1 Fig — (TIF) [file pone.0257218.s001.tif]

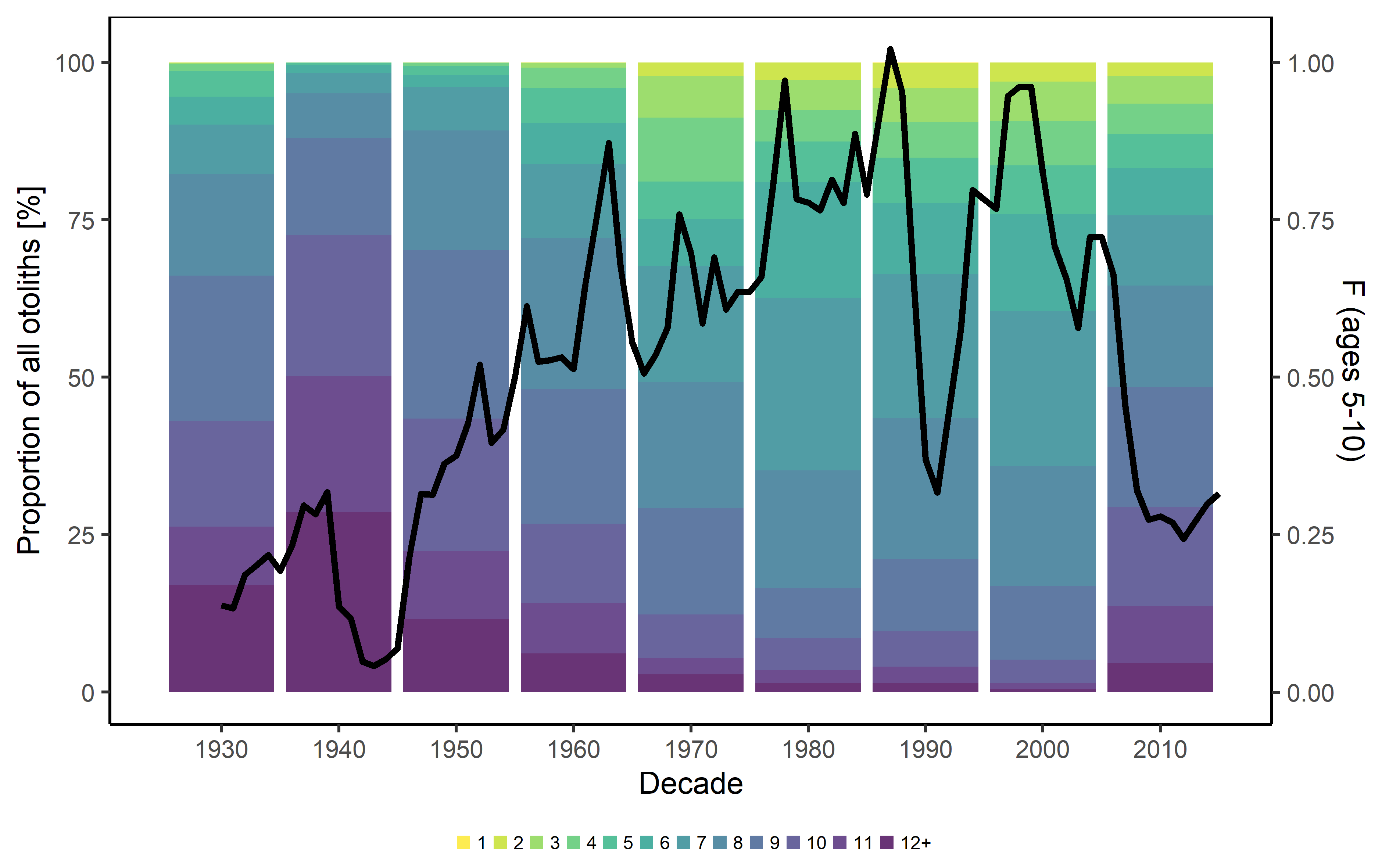

Supplement: S2 Fig — Thick black line represents the average fishing mortality from ages 5 to 10. (TIF) [file pone.0257218.s002.tif]

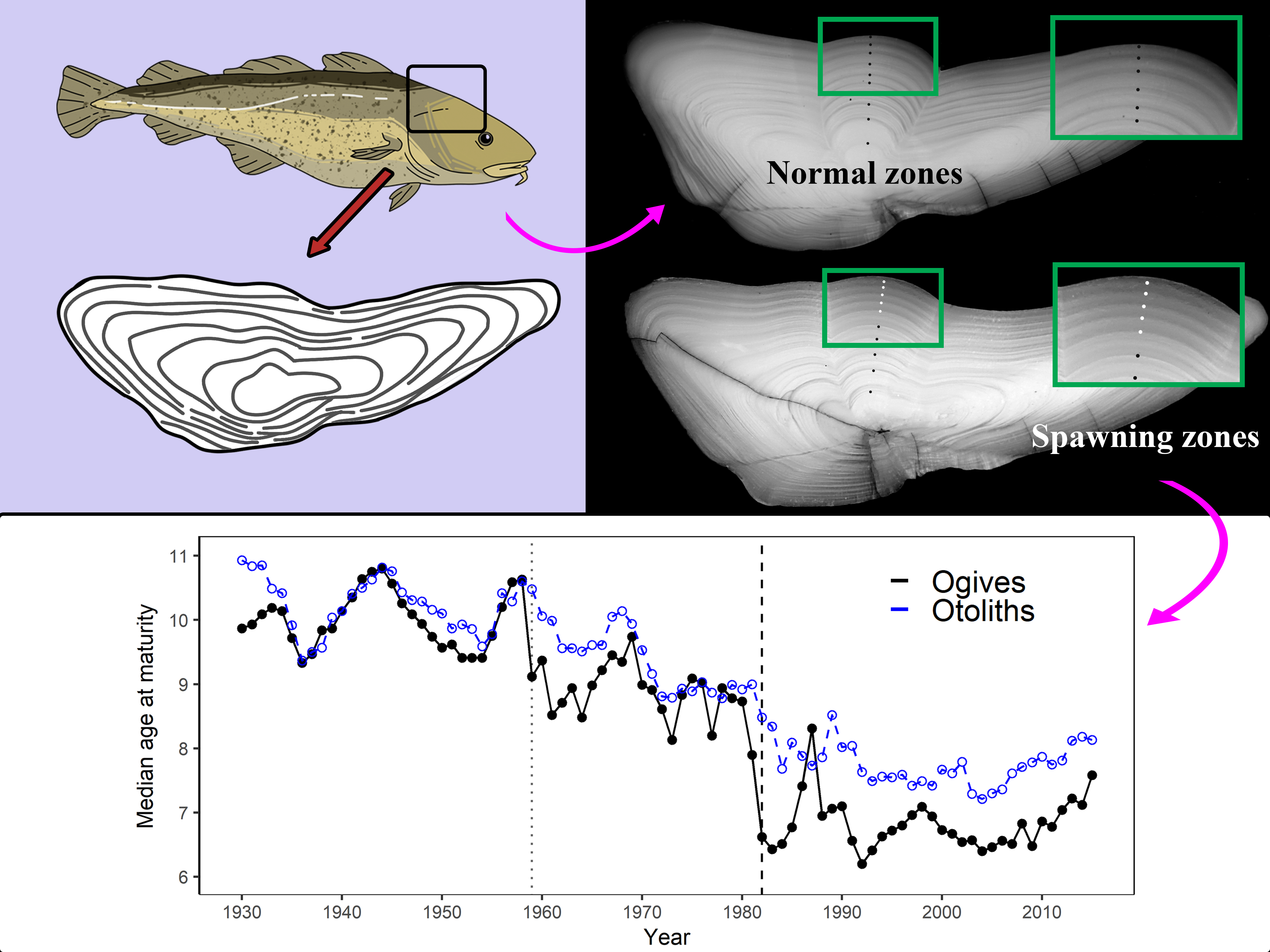

Supplement: S1 Graphical abstract — (TIF) [file pone.0257218.s004.tif]
