# Supplementary material for: Otolith “spawning zones” across multiple Atlantic cod populations: Do they accurately record maturity and spawning?
Source: PLoS One. 2021 Sep 13;16(9):e0257218. doi: 10.1371/journal.pone.0257218 (PMC8437307; doi:10.1371/journal.pone.0257218)
Supplement: S1 Table — Total of 41 samples were randomly selected across all 6 cod populations and read blindly in three individual instances by each reader. Median number of spawning zones found for each sample by each reader were then compared. (DOCX) [file pone.0257218.s003.docx]

**S1 Table.** **Summary statistics of reader precision and agreement for spawning zone interpretation.** A total of 41 otolith images were randomly selected across all 6 cod populations and read by two readers. Each reader read the age and number of spawning zones blindly on three separate occasions. The median number of spawning zones identified across the three readings for each sample by each reader were then compared.

| Test | N | N samples with at least one different reading | Percentage agreement | Average coefficient of variation | Average standard deviation |
| --- | --- | --- | --- | --- | --- |
| Internal consistency reader A | 41 | 4 | 90.2% | 7.3% | 0.06 |
| Internal consistency reader B | 41 | 10 | 75.6% | 22.1% | 0.18 |
| Median A vs median B | 41 | 12 | 70.7% | 24.1% | 0.33 |
