# Supplementary material for: Otolith “spawning zones” across multiple Atlantic cod populations: Do they accurately record maturity and spawning?
Source: PLoS One. 2021 Sep 13;16(9):e0257218. doi: 10.1371/journal.pone.0257218 (PMC8437307; doi:10.1371/journal.pone.0257218)
Supplement: Attachment — Submitted filename: Denechaud_Response_to_reviewers.docx [file pone.0257218.s005.docx]

**Côme Denechaud^1,2,*^**, Audrey J. Geffen^1,2^, Szymon Smoliński^1,3^, Jane A. Godiksen^1^

^1^Demersal Fish Research Group, Institute of Marine Research (HI), Bergen, Norway

^2^Department of Biological Sciences, University of Bergen (UiB), Norway

^3^Department of Fisheries Resources, National Marine Fisheries Research Institute, Gdynia, Poland

Dear editor and reviewer,

We are very grateful for the positive reception and valuable comments provided in your review of our manuscript *Otolith “spawning zones” across multiple Atlantic cod populations: do they accurately record maturity and spawning?* We have carefully reviewed these suggestions and revised our manuscript accordingly. Our answers to each point are provided below with the corresponding changes in the manuscript (“Denechaud_Revised_manuscript_track_changes.docx”).

**Reviewer: 1**

This is an interesting manuscript that pulls together a huge amount of data to take a comprehensive look at a phenomenon referred to as spawning zones in Atlantic cod otoliths. Recent experimental work by others demonstrated that the formation of these marks does not necessarily correspond to the onset of spawning in cod. The present manuscript addresses this by comparing spawning marks from populations across most of the north Atlantic and testing them against direct measures of maturation status (gonadal examination). There are interesting trends decadally as well as regionally. One interesting conclusion is that spawning marks are a species trait, and rather than corresponding exactly to spawning, may rather reflect a "settling down" of the energy balance in a fish following maturity. Reasoned arguments are made in the Discussion about why these marks may occur, even when fish skip spawning to instead feed and regain energy.

One question I have is whether the authors can suggest why the translucent zones maintain their relative thicknesses, while the opaque zones become reduced. Yes, it is an energy balance, but what is it that causes no change in the translucent zones.

**The spawning zones we describe here are characterized by being narrow and regular, as well as having proportionally much thinner opaque zones in comparison to the inner increments. In the manuscript we suggest that the formation of these spawning zones is likely tied to a profound shift in energy allocation from growth toward reproduction. A direct result is that the mature fish grow little even during the normal high-growth periods, and the resulting opaque zones become much narrower.**

**For the vast majority of fish species, the alternance of thick opaque and thin translucent zones is usually explained by a natural seasonal variability in somatic growth and metabolic activity when environmental conditions (i.e. temperature, resource availability, etc) become less optimal and therefore little material, also much less organic in nature, is biomineralized in the otolith. Biomineralization models for cod otoliths suggest that opaque zones are the result of increased otolith protein matrix synthesis (providing more sites for biomineralization) which mirror higher somatic growth in the fish. As somatic growth decreases – either from allocation of energy to reproduction, or changes in environmental conditions (primarily temperature – too high or too low) – the rate of matrix production reduces and biomineralization rates decline and the resulting optical characteristic is the translucent zone.**

**The translucent zone in Barents Sea cod begins forming in October, at a point when gonad maturation is underway. The opaque zone starts appearing in April after spawning and grows throughout the summer months. It is this zone that is most affected/flexible in response to fish condition. However, in North Sea cod and other populations living closer to the thermal maximum, the translucent zone is triggered by high temperatures toward the end of the summer, and opaque zones form in cooler months of winter and spring. Both of these patterns suggest that there is more scope for response to conditions in the opaque zone compared to the translucent zones. Given this suspected mechanism, we suggest that translucent zones remain relatively similar in spawning zones because they are not affected by the change of energy allocation in the first place. If immature fish form translucent zones when their somatic growth is reduced due to seasonal environmental and metabolic changes, adult fish may undergo the same seasonal cycle even after the energy shift following maturity, i.e. mature fish grow less (and biomineralize comparatively less otolith material) than immature fish during the high-growth periods due to a shift in energy balance (= smaller opaque zones), but both still experience little to no growth the rest of the time (= translucent zones).**

**As we report in the manuscript, the translucent part of spawning zones is often even comparatively thicker and darker which could indicate that the “no-growth” period for mature fish may be longer and even more pronounced, with very little organic materials being mineralized in the otolith.**

**References:**

**Fablet R, Pecquerie L, de Pontual H, Hoie H, Millner R, Mosegaard H, et al. Shedding Light on Fish Otolith Biomineralization Using a Bioenergetic Approach. Plos One. 2011;6(11). doi: 10.1371/journal.pone.0027055**

**Hoie H, Folkvord A, Mosegaard H, Li L, Clausen LAW, Norberg B, et al. Restricted fish feeding reduces cod otolith opacity. Journal of Applied Ichthyology. 2008;24(2):138-43. doi: DOI 10.1111/j.1439-0426.2007.01014.x**

**Hoie H, Millner RS, McCully S, Nedreaas KH, Pilling GM, Skadal J. Latitudinal differences in the timing of otolith growth: A comparison between the Barents Sea and southern North Sea. Fish Res. 2009;96(2-3):319-22. doi: DOI 10.1016/j.fishres.2008.12.007**

**Skjæraasen JE, Nash RDM, Korsbrekke K, Fonn M, Nilsen T, Kennedy J, et al. Frequent skipped spawning in the world's largest cod population. P Natl Acad Sci USA. 2012;109(23):8995-9. doi: 10.1073/pnas.1200223109.**

**Folkvord A, Jorgensen C, Korsbrekke K, Nash RDM, Nilsen T, Skjaeraasen JE. Trade-offs between growth and reproduction in wild Atlantic cod. Can J Fish Aquat Sci. 2014;71(7):1106-12. doi: DOI 10.1139/cjfas-2013-0600.**

Minor edits:

- On line 225 the authors write "over 380 000 NEA cod" whereas everywhere else they write the exact number (382 218). Suggest they use the latter throughout.

**Changed to the precise number for clarity.**

- Line 243: insert "calendar" between "each" and "year"

**Added.**

- Lines 305-308, Figure 4 caption: please explain what the numbers on the columns refer to.

**Added a sentence to the caption to describe what the numbers in the columns are (L306-307)**

- Line 315 and elsewhere: "arcing shape" may not be the best descriptor. Consider using "convex" instead.

**Changed “arcing” to “convex” in all occurrences, L316 and L356**

Overall, the authors are to be congratulated on such a comprehensive study.

**We are very grateful for the positive reception of our study.**
